# Efficient Enzymatic Hydrolysis of Biomass Hemicellulose in the Absence of Bulk Water

**DOI:** 10.3390/molecules24234206

**Published:** 2019-11-20

**Authors:** Shaghayegh Ostadjoo, Fabien Hammerer, Karolin Dietrich, Marie-Josée Dumont, Tomislav Friscic, Karine Auclair

**Affiliations:** 1 Department of Chemistry, McGill University, 801 Sherbrooke Street West, Montreal, Quebec, H3A 0B8, Canada; shaghaayegh@gmail.com (S.O.); fabien.hammerer@gmail.com (F.H.); 2 Bioresource Engineering Department, McGill University, 21111 Lakeshore Road, Ste-Anne de Bellevue, Quebec, H9X 3V9, Canada; karolin.dietrich@mail.mcgill.ca (K.D.); marie-josee.dumont@mcgill.ca (M.-J.D.)

**Keywords:** hemicellulose, xylan, hemicellulase, xylanase, mechanochemistry, mechanoenzymology, solvent-free, glycosyl hydrolase, saccharification, biowaste

## Abstract

Current enzymatic methods for hemicellulosic biomass depolymerization are solution-based, typically require a harsh chemical pre-treatment of the material and large volumes of water, yet lack in efficiency. In our study, xylanase (E.C. 3.2.1.8) from *Thermomyces lanuginosus* is used to hydrolyze xylans from different sources. We report an innovative enzymatic process which avoids the use of bulk aqueous, organic or inorganic solvent, and enables hydrolysis of hemicellulose directly from chemically untreated biomass, to low-weight, soluble oligoxylosaccharides in >70% yields.

## 1. Introduction

The depletion of petroleum resources and increasing awareness of the impact of the petrochemical industry on the environment have motivated research efforts aiming to exploit sustainable feedstocks, including biowaste valorization. Lignocellulosic biomass is the most abundant biowaste, with >10^12^ tons generated annually [1], and has been proposed as a sustainable fossil fuel replacement alternative [2]. The efficient deployment of biomass-based technologies is however challenged by the highly inert nature of its polymeric constituents [3], the breakdown of which is a necessary step to the exploitation of biomass as a resource [2].

Vascular plant biomass is mainly composed of cellulose, hemicellulose, and lignin. At 20–30% of the total dry weight of plants, hemicellulose is the second most abundant biopolymer on Earth [4]. Xylan, the main type of hemicellulose, is a vast group of branched heteropolysaccharides, which principally consists of β-1,4-linked xylose units, with side branches of α-arabinofuranose, α-glucuronic acids, or other monosaccharides [2,5].

The chemical degradation of biomass xylans produces mainly xylose and xylooligosaccharides [6]. It is usually achieved by exposing the biomass to aggressive chemicals such as acids or bases at high temperature and pressure, with the generation of undesirable side products [7,8,9,10], which are preferably removed before further processing [11,12,13].

Alternatively, numerous bacteria and fungi have naturally evolved to efficiently use lignocellulosic biomass as a source of nutrients [14]. These microorganisms are incapable of transporting the large biopolymers inside the cells. Instead, they secrete a mixture of enzymes, which work at the interface between the solid biomass and moist air to break down the polymers into small soluble oligomers and monomers that are next transported inside the cell and metabolized [8]. The xylan component is degraded under ambient conditions by hydrolytic enzymes called xylanases [15,16].

Whereas in vitro hydrolysis of xylans by xylanases in solution has been demonstrated, this strategy necessitates harsh chemical pre-treatment of the biomass in order to expose the hemicellulose, large volumes of water to create a suspension or slurry, and still suffers from low hydrolysis rates and turnover numbers [17,18,19,20,21].

Recent studies by our group, and others [22,23,24,25,26,27,28,29,30,31,32], have established that enzymes can function very well without bulk organic or aqueous solvent. In particular, our group has demonstrated that in the absence of bulk water, cellulases can efficiently catalyze cellulose hydrolysis to glucose [29,30], and chitinases can favor the clean depolymerization of chitin to *N*-acetylglucosamine [31]. Our unconventional approach combines techniques from solvent-free mechanochemistry [33], such as ball milling [34,35,36] and accelerated aging [37,38], with enzymatic catalysis. While mechanical processing of lignocellulose has been used for decades [39,40], the idea that gentle ball milling can promote enzyme activity is recent and has shown great potential. In contrast to the established methods in enzymology, we report here that xylanase can be more efficient in the absence of bulk aqueous solvent. This is consistent with the natural environment of xylanases, and provides an innovative, efficient way to hydrolyze xylans from biomass directly, without needing chemical pre-treatment or bulk water, thus generating minimal waste. Instead of using bulk water, the herein presented methodology operates under conditions resembling liquid-assisted grinding (LAG), [41,42,43] in which reactant solubility is not a significant factor and where minuscule amounts of a liquid phase are utilized to accelerate or direct chemical transformations.

## 2. Results and Discussion

We opted to use xylanase from *Thermomyces lanuginosus* (E.C. 3.2.1.8) because it is available commercially as a lyophilized powder, mostly as a single protein (which was confirmed by electrophoresis, Figure S1) and is stable at temperatures up to 80 °C [44]. The commercial powder was found to have an extremely low protein content (0.4% *w*/*w* measured by classical Bradford assay), with the rest of the mixture likely consisting of buffer salts, lyoprotectants, and/or growth medium. In the remainder of this manuscript, enzyme loading will be described as the mass of protein per total initial solid mass used, i.e., the combined mass of the substrate and of the enzyme preparation. The enzymatic reactions reported herein proceed either via ball milling with or without subsequent aging (static incubation) at 55 °C, or via multiple cycles of milling and aging—a process known as reactive aging (RAging) [29,30,31,32]. Unless mentioned otherwise, milling was performed at 30 Hz and room temperature, in 10 mL teflon jars containing two stainless steel balls (7 mm in diameter). A variety of substrates were investigated, including purified xylans from birchwood and oat spelt, as well as raw sugarcane bagasse and wheat straw biomass.

As a hydrolytic enzyme, xylanase catalyzes the reaction between xylans and water. Herein, except for control reactions in solution (Table 1), water is present in amounts too low to be considered a bulk solvent (moist solid mixture), playing the role of a substrate and also additive that accelerates mechanochemical reactions. The amount of water is conveyed as a ratio of the volume of liquid added in μL, to the weight of all solids used in mg, and abbreviated as η [42]. The optimal η for xylanase was found to be 0.6 µL/mg with purified xylans, and 1 µL/mg with raw biomass (Figures S2 and S3), which correspond to initial mixtures with the consistence of sticky solids. Our enzymatic reactions are therefore defined as liquid-assisted (defined as η < 2) [42], or solvent-less rather than solutions or suspensions.

Consistent with the established behavior of enzymes in solution, the percent hydrolysis of xylans observed after milling only, or after milling followed by aging, was found to raise with an increase in enzyme loading, and the optimal value differed with each substrate (Figure S4). Reducing the milling frequency from 30 Hz to 10 Hz was detrimental to the yield, with a 5% decrease when milling only, and a 20% lower yield when milling is followed by aging (Figure S5). This is most likely due to poorer sample mixing. All subsequent experiments were performed at 30 Hz.

**Figure 1:**
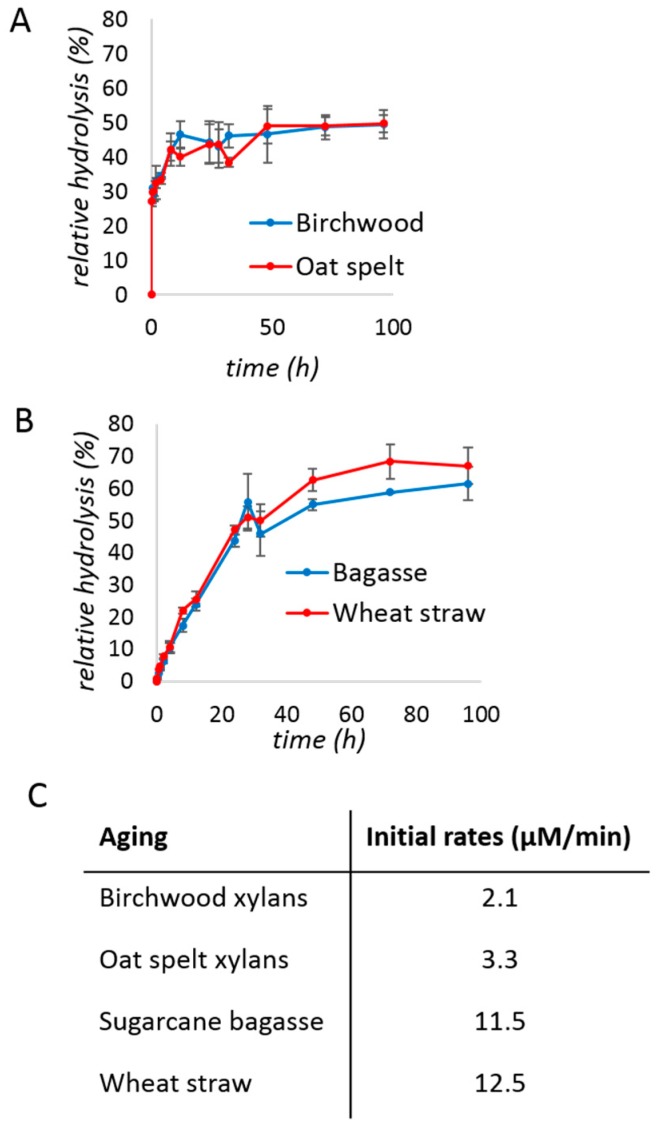
Studies of xylanase kinetics under aging conditions with: (**A**) birchwood (blue) and oat spelt (red) xylans; (**B**) sugarcane bagasse (blue) and wheat straw (red). The reaction mixtures of (**A**) contained 200 mg of xylan, 50 mg of the commercial xylanase mixture (i.e., 0.2 mg of protein or 0.08% *w*/*w*), and 150 µL of water. The mixtures of (**B**) contained 400 mg of substrate, 200 mg of the commercial xylanase mixture (i.e., 0.8 mg of protein or 0.13% *w*/*w*), and 600 µL of water. The reaction was milled for 5 min, before transferring to an incubator at 55 °C. (**C**) Initial rates calculated from the data of panels A and B. The *t* = 0 background was subtracted. Reactions were performed in triplicates and the error bar is the standard deviation.

Kinetic studies of the xylanase (0.08% protein loading *w*/*w*) were performed after a brief period of milling (5 min) followed by aging, with regular sampling over time. The hydrolysis yield was approximated using the 3,5-dinitrosalicylic acid (DNS) assay [45] and plotted over time. With purified xylan substrates (Figure 1A), a typical hyperbolic behavior was obtained, reaching a plateau after ca. 20 h, at approximately 50% hydrolysis. The initial rate of the reaction was 2–3 µM/min (Figure 1C).

We next turned to substrates directly obtained from the agricultural industry: Raw sugarcane bagasse and wheat straw samples of known xylan content (19.9% and 22.3%, respectively). Both substrates were milled briefly for 10 min before use, in order to reduce particle size below 1 mm. They were used directly without chemical pre-treatment. The depolymerization of biomass (Figure 1B) during aging after milling for 5 min, proceeded with a higher initial rate (11–12 µM/min) and to a better yield (>60%) than with purified xylans.

**Figure 2:**
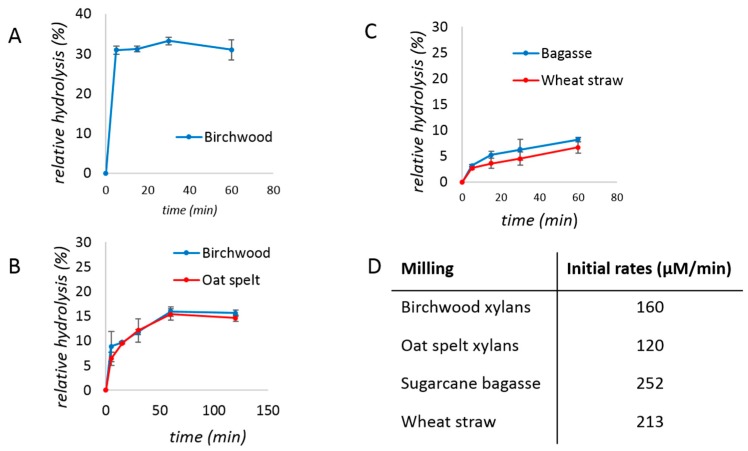
Studies of xylanase reaction kinetics under milling for (**A** and **B**) birchwood (blue) and oat spelt (red) xylans, or (**C**) sugarcane bagasse (blue) and wheat straw (red). (**A**) the reaction mixture contained 200 mg of birchwood xylan, 500 mg of the commercial xylanase mixture (i.e., 0.2 mg of protein or 0.1% *w*/*w*), and 150 µL of water. (**B**) the mixtures contained 200 mg of birchwood or oat spelt xylan, 10 mg of the commercial xylanase mixture (i.e., 0.04 mg of protein or 0.02% *w*/*w*), and 150 µL of water. (**C**) the reaction mixtures contained 400 mg of substrate, 20 mg of the commercial xylanase mixture (i.e., 0. 0.08 mg of protein or 0.02% *w*/*w*), and 600 µL of water. (**D**) Initial rates calculated for the reactions of panels B and C. Reactions were performed in triplicates and the error bar is the standard deviation.

Under milling only, the xylanase-catalyzed cleavage of birchwood or oat spelt xylans over time was very fast and plateaued rapidly (Figure 2A). As a result, the enzyme loading had to be lowered to 0.02% protein (*w*/*w*) to allow detection of the initial time points. The observed kinetic behavior was again hyperbolic (Figure 2B), but as expected for conditions with less enzyme, the reactions leveled off at lower yields (<20%). Based on the initial rates measured, biomass was again a better substrate than purified xylans during milling (Figure 2C,D). Interestingly the initial rate was found to be higher during milling (Figure 2D) than during aging (Figure 1C), but reached a plateau sooner.

**Figure 3:**
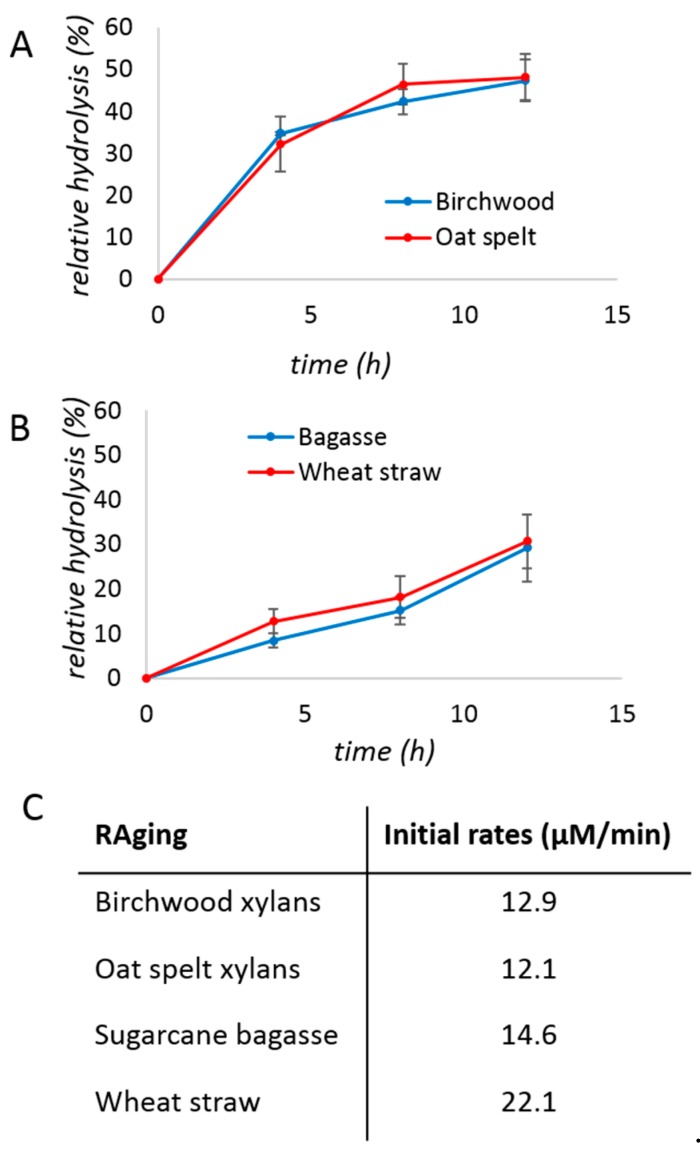
Studies of xylanase reaction kinetics under RAging conditions with (**A**) birchwood (blue) or oat spelt (red) xylans, or (**B**) sugarcane bagasse (blue) or wheat straw (red), and the corresponding initial rates (**C**). (**A**) the reaction mixtures contained 200 mg of xylan, 50 mg of the commercial xylanase mixture (i.e., 0.2 mg of protein or 0.08% *w*/*w*), and 150 µL of water. (**B**) the reaction mixtures contained 400 mg of substrate, 100 mg of the commercial xylanase mixture (i.e., 0.4 mg of protein or 0.08%), and 600 µL of water. The reactions were milled for 5 min, before incubating at 55 °C for 55 min, and repeating this cycle up to 12 times. (**C**) Initial rates calculated for the reactions of panels A and B. Reactions were performed in triplicates and the error bar is the standard deviation.

In previous studies of cellulose digestion by cellulases, it was found that RAging allowed significant improvement of both the rate and the turnover number compared to milling and aging only once [22]. The activity of xylanase (0.08% protein *w*/*w*) on xylans under RAging conditions, i.e., by repeating 12 one-hour cycles consisting of milling for 5 min and aging for 55 min, again exhibited a hyperbolic profile, with initial rates of 12–13 µM/min, and reached ca. 50% yield in 12 h (Figure 3A,C). The saccharification of biomass during RAging (Figure 3B) was also slower than when milling once and aging. Overall, these results suggest that the optimal milling regime is not universal across different enzymes.

**Figure 4:**
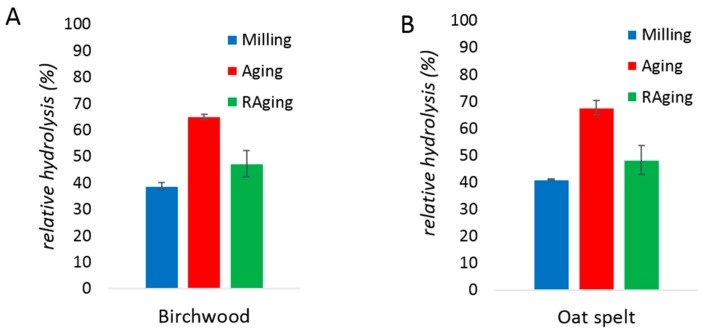
Progress of enzymatic hydrolysis of birchwood xylan (**A**) and oat spelt xylan (**B**) by xylanase after 30 min of milling (blue), 5 min of milling followed by 72 h of aging at 55 °C (red), or 12 h of RAging via 12 cycles of 5 min milling + 55 min aging (green). The reactions were performed at 0.08% *w*/*w* protein loading on 200 mg of xylan substrate, with 150 µL of water (η = 0.6 µL/mg). Reactions were performed in triplicates and the error bar is the standard deviation.

Regular sample collection to monitor the reaction may affect reproducibility and progress by altering the temperature and total volume of the reaction mixture in the milling jar over time. The next series of experiments were therefore focused on end-point hydrolysis yields. Reactions using a protein loading of 0.08% (*w*/*w*) revealed that neither 30 min of milling only, nor 12 h of RAging (i.e., 12 cycles of repeating a sequence of 5 min milling and 55 min aging), resulted in more than 50% hydrolysis (Figure 4). The results were similar for both birchwood or oat spelt xylans substrates. Interestingly, treating the xylanase reaction mixture to a 30 min milling period followed by aging for 72 h hydrolyzed the xylans with ca. 70% yield (Figure 4). Taken together, these results suggest that xylanase tolerates well short milling periods and can work efficiently for days under aging conditions.

**Figure 5:**
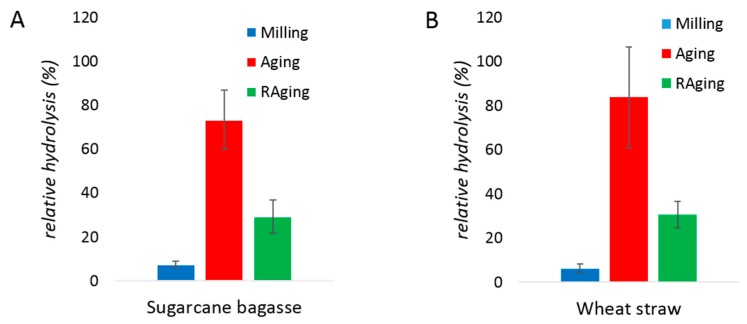
Enzymatic hydrolysis of sugarcane bagasse (**A**) and wheat straw (**B**) by xylanase after 30 min of milling (blue), 30 min of milling and 72 h of aging at 55 °C (red), or 12 h of RAging via 12 cycles of 5 min milling + 55 min aging (green) conditions. The reactions were performed at 0.13% (*w*/*w*) protein loading on 400 mg of substrate, with 600 µL of water (η = 1 µL/mg). Reactions were performed in triplicates and the error bar is the standard deviation.

With biomass, enzymatic hydrolysis after 30 min of milling resulted in 12% and 8% yields from sugarcane bagasse and wheat straw, respectively (Figure 5). The conversion during RAging (12 one-hour cycles of milling for 5 min and aging for 55 min) reached hydrolysis yields of 38% and 36%, for the two biomass samples respectively. Similar to the results obtained with purified xylans, milling followed by aging was more efficient, with yields of 73% and 84% obtained for sugarcane bagasse and wheat straw hydrolysis, respectively. Importantly, such high conversions are extremely unusual for xylanase enzymes non-supplemented with cellulases, especially on untreated biomass. Overall, whether the processing method was milling only, a single cycle of milling and aging, or RAging, the initial rate of hydrolysis was lower with purified xylans than with biomass (Figure 1, Figure 2 and Figure 3), and the enzyme was also active for a longer period of time with biomass, reaching higher yields.

**Figure 6:**
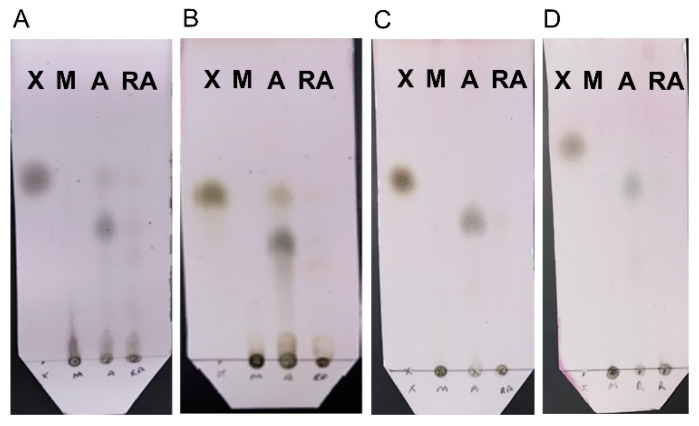
Thin Layer Chromatography (TLC) analysis of the soluble products of enzymatic reactions after 30 min milling (M), 30 min milling and 72 h aging at 55 °C (A), or 12 h RAging via 12 cycles of 5 min milling followed by 55 min aging (RA), starting from either 200 mg of birchwood (Panel **A**) or oat spelt (Panel **B**) xylans hydrolyzed using 0.13% *w*/*w* protein loading at η = 1 µL/mg, or 400 mg of sugarcane bagasse (Panel **C**) or wheat straw (Panel **D**) biomass hydrolyzed with 0.08% *w*/*w* protein at η = 0.6 µL/mg. Lane X was loaded with the monosaccharide standard xylose.

Chemical analysis of the reaction mixtures to identify the soluble products revealed that, for all substrates, milling and RAging produce mostly oligomers (Figure 6). In contrast, milling and aging only once was found to yield predominantly the xylose monosaccharide and/or disaccharide. Sugar analysis was also performed to quantify the amount of xylose monomer produced (Table S1). When compared to total reducing end yields (DNS assay), the data confirms that xylose is a minor product, although highest when milling and aging only once, with soluble oligomers being the major product in all cases. Thus, xylanase is not only more efficient under milling followed by aging, compared to either milling or RAging, but it also generates a cleaner product. This contrasts with the activity of cellulases which was found to be optimal under RAging conditions [29] and suggests that even enzymes of the same family like cellulases and xylanases, both of which are glycoside hydrolases, can have different preferences for optimal activity in the absence of bulk water.

Finally, we compared equivalent xylanase reactions with and without bulk water. Both reactions were performed at the same substrate/enzyme ratio, protein content, temperature, and duration, and only differ in the amount of water added. The results (Table 1) are consistent with a larger volume of water (considered standard conditions) being detrimental to xylanase activity. With xylans, the hydrolysis yield dropped by approximately three fold when compared to either 30 min of milling, or milling followed by 72 h of aging. The difference was most striking with untreated biomass allowed to react for three days, with the yield dropping from ca. 79% to ca. 5% with added bulk water. These results strongly suggest that solvent-less conditions can offer a more suitable platform for optimal performance of xylanase.

## 3. Materials and Methods

### 3.1. Substrates, Enzyme, and Equipment

Birchwood xylan (≥90%, now discontinued) and oat spelt xylan (≥70%, now discontinued) were purchased from Sigma-Aldrich (Missouri, MO, USA). The untreated sugarcane bagasse and wheat straw samples were provided and their contents analyzed by Iogen Corporation (Ottawa, Canada). Xylanase from *T. lanuginosus* was purchased as a lyophilized powder of 2798 U/g from Sigma-Aldrich (Missouri, MO, USA). Analysis of this commercial preparation using the Bradford assay revealed a protein content of only ~0.4%, with the rest likely consisting of buffer, lyoprotectants and/or growth medium. The sugars contained in this matrix were quantified using the DNS assay and this amount was subtracted from reaction product measurements. All the reagents used in experimental section were of practical grade. Ball milling was conducted in a FTS 1000 shaker mill from Form Tech Scientific (Montreal, QC, Canada). Absorption was measured on a plate reader SpectraMax i3x from Molecular Device (San Jose, CA, USA).

### 3.2. SDS-Page Gel Analysis

Denatured protein solution (15 µL of a 4 mg/mL solution in 50 mM sodium acetate buffer, pH 5, boiled for 10 min) containing an elution marker was deposited in a well of a pre-cast gel Miniprotean TGX 4–20% acrylamide from BIO-RAD. The SDS-PAGE was run for ca. 40 min with a constant voltage of 150 V in a Tris/Glycine/SDS buffer. After completion, the gel was stained with a Coomassie blue (0.25% *w*/*v*) solution in water/MeOH/AcOH (5:4:1 *v*/*v*/*v*) for 15 min. It was then de-stained using a mixture of water/MeOH/AcOH (5:4:1 *v*/*v*/*v*) for one hour. It was further de-stained overnight in water. Protein weight are characterized compared to a protein ladder (LMW calibration kit from GE).

### 3.3. Relative Hydrolysis

The reaction yields were first estimated using the classical dinitrosalicylic acid method [40]. DNS reacts with the reducing end of sugars and does not discriminate between them. Detailed sugar analysis (see Section 3.4) was performed on key samples to determine specific xylose concentrations and yields. The DNS reagent was prepared according to Miller [42] with the following modifications: DNS (1 g), potassium sodium tartrate (30 g), and water (50 mL), and NaOH (20 mL of a 2 M solution) were mixed before diluting to 100 mL in water. The solution was filtered through cotton and stored at 4 °C for up to one month. The DNS solution was calibrated using D-xylose dissolved in water over a range of 0.2 to 1.0 mg/mL. For measuring the concentration of reducing sugars, aliquots (~15 mg) were suspended in ice cold water to a final xylan concentration of 10 mg/mL as determined by the Equation (1):

*V* = 0.1 × *m*_xylan/_(*m*_total_ × *m*_sample_) (1)

The *m*_xylan_ is defined as the mass of xylan that was added to the milling jar, the mass_total_ is defined as the total mass of all reagents (water included) added into the milling jar, and the m_sample_ is the mass of the aliquot taken from the milling jar. V is defined as the volume of water that should be added to reach a final xylan concentration of 10 mg/mL. The microcentrifuge tubes containing the suspension of the mixture from the solvent-free reactions in ice-cold water were immediately placed into a boiling water bath for 30 min to inactivate the enzymes. For in-solution enzymatic reactions, aliquots collected in a microcentrifuge tube were directly boiled by immersion in a boiling water bath. Once cooled, the samples from the solvent-free reactions were disturbed and broken down to small pieces with a spatula and centrifuged at 21,100× *g* for 5 min to pellet any insoluble material. The supernatant was used to measure the amount of reducing sugar using the DNS reagent. The supernatant (200 µL, diluted 4×) was added to the DNS solution (100 µL), and was boiled for 5 min. Then, 200 µL was transferred into the well of a clear-bottom 96-well microtiter plate. Absorbance was measured at 540 nm using a Molecular Devices SpectraMax i3x (San Jose, California, USA) microplate reader with pathcheck enabled. Yields were calculated on a dry mass basis by approximating hemicellulose to linear infinite chains of linked xylose.

### 3.4. Sugar Analysis

The monosaccharides were analyzed in a sugar analyzer (YSI 2900, Yellow Spring Instruments (Yellow Spings, OH, USA). Lyophilized pellets were weighed (1–12 mg) and suspended in distilled water (100 µL). The samples were vortexed for 30 s, kept at 30 °C for 30 min to allow for full sugar dissolution, and vortexed again for 30 s. Next, the mixtures were centrifuged for 5 min at 9391× *g*, and the supernatant was separated and analyzed. This analyzer uses glucose and xylose specific membranes, and calibrates itself automatically with standard glucose (2.5 g/L) and xylose (20 g/L) solutions. The pH was kept at 7.0 by using a mono and dibasic sodium phosphate buffer (YSI 2357, 0.1 M, pH 7.0).

### 3.5. Pre-Milling of Biomass

1.5 g of raw sugarcane bagasse or wheat straw was introduced in a 30 mL stainless steel milling jar with two stainless steel balls of 10 mm diameter. The jar was then shaken at 30 Hz for 10 min to afford a fine powder which was kept in room conditions.

### 3.6. Determination of Optimal Water Loading

Birchwood and oat xylan (200 mg), and xylanase (50 mg of the commercial preparation) were introduced into a 10 mL volume teflon jar, along with two stainless steel balls of 7 mm diameter each. Then, 50 µL, 100 µL, 150 µL, or 200 µL water was added to the mixture before milling at 30 Hz and room temperature for 30 min. The samples were analyzed for reducing end sugars using the DNS protocol described above.

With sugarcane bagasse or wheat straw, the substrate (400 mg), xylanase (200 mg of the commercial preparation) were added to a 10 mL volume teflon jar, along with two stainless steel balls of 7 mm diameter each. Then, 360 µL, 600 µL, or 720 µL of water was added to the mixture before milling at 30 Hz and room temperature for 30 min. The samples were analyzed for reducing end sugars using the DNS protocol described above.

### 3.7. TLC Analysis of the Soluble Enzymatic Reaction Products

TLC analysis was performed with 60 Å silica gel TLC plates (F-254) from Silicycle, using chloroform/acetic acid/H_2_O (6:7:1) as the eluent. The eluted TLC plates were stained with a solution of *p*-anisaldehyde (15 g of *p*-anisaldehyde, 250 mL of ethanol and 2.5 mL of conc. sulfuric acid), followed by heating with a blow dryer until completely dry.

### 3.8. Reactions in Solution

Birchwood and oat xylans (100 mg) and xylanase (25 mg of the commercial preparation) were mixed in water (10 mL) in a 50 mL tube. With sugarcane bagasse or wheat straw, the substrate (100 mg) and xylanase (50 mg) were mixed in water (10 mL) in a 50 mL tube. Each reaction was allowed to proceed on a rotary shaker (400 rpm) at room temperature for 30 min, in order to match the conditions of solvent-free milling, or at 55 °C for 72 h to match the conditions of solvent-free milling and aging. Reaction aliquots were stored at −20 °C until analysis.

## 4. Conclusions

Many attempts have been made over the past decades to reduce the cost and increase the efficacy of enzymatic hemicellulose hydrolysis [46]. These efforts have mainly focused on identification and engineering of novel enzymes [19,21,47], or accessory proteins [3,48]. In parallel, researchers have also worked at optimizing biomass pre-treatment methods to improve enzymatic reaction yields [49]. We propose here a starkly different strategy inspired from the natural environment of xylanases. Our unconventional method consists of briefly milling a mixture of lyophilized enzyme preparation, solid substrate, and several equivalents of water, before allowing the resulting moist solid to sit under controlled conditions (i.e., aging). Besides improving the yields compared to the same enzymatic reaction performed in dilute buffer, this unique process does not require any harsh chemicals, and greatly decreases the overall reaction volume, therefore facilitating handling of the mixture, and generating significantly less waste. Importantly, this enzymatic method proceeds with yields >70% at very low protein loading (0.08% *w*/*w*), without the need for biomass pre-treatment.

## Figures and Tables

**Table 1:** Comparison of the percent hydrolysis yield (based on the DNS assay) of xylanase reactions on different substrates in the presence or absence of bulk water. In both cases, 100 mg of substrate was used, with 125 µL or 10 mL of water, and xylanase at a protein loading of 0.08% *w*/*w*.

	No Bulk Water		With Bulk Water	
	Milling 30 min, RT	Aging 72 h, 55 °C	Shaking 30 min, RT	Shaking 72 h, 55 °C
**Birchwood xylan**	39 ± 1%	65 ± 1%	14 ± 1%	24 ± 3%
**Oat spelt xylan**	41 ± 1%	68 ± 3%	14 ± 1%	26 ± 1%
**Sugarcane bagasse**	12 ± 6%	73 ± 14%	1.6 ± 0.5%	4 ± 1%
**Wheat straw**	8 ± 4%	84 ± 23%	2.4 ± 0.2%	5 ± 2%

## Supplementary Materials

The following are available online at https://www.mdpi.com/1420-3049/24/23/4206/s1, Figure S1: SDS-PAGE analysis (0.1% SDS (w/v), 25 mM Tris) of commercial xylanase from Thermomycec lanuginosus., Figure S2: Optimization of the amount of water (reported as η, i.e., the volume of water added over the total amount of solid in µL/mg) for the hydrolysis of purified birchwood (A) or oat spelt (B) xylan by xylanase under milling conditions., Figure S3: Optimization of the amount of water (reported as η, i.e., the volume of water added over the total amount of solid in µL/mg) for the hydrolysis of sugarcane bagasse (A) or wheat straw (B)., Figure S4: Optimization of the enzyme loading for the hydrolysis of birchwood xylan, oat spelt xylan, sugarcane bagasse or wheat straw by xylanase under milling only (30 min) or milling (30 min) followed by aging (72 h at 55 °C)., Figure S5: Effect of milling frequency (10 Hz or 30 Hz) on the xylanase-catalyzed hydrolysis of oat spelt xylan. Table S1: Results of sugar analyses.
