# Supplementary material for: Efficient Enzymatic Hydrolysis of Biomass Hemicellulose in the Absence of Bulk Water
Source: Molecules. 2019 Nov 20;24(23):4206. doi: 10.3390/molecules24234206 (PMC6930478; doi:10.3390/molecules24234206)
Supplement: Supplementary file 1 [file molecules-24-04206-s001.pdf]

Electronic Supporting Information

Efficient enzymatic hydrolysis of biomass hemicellulose in the absence of bulk water

Shaghayegh Ostadjoo, Fabien Hammerer, Karolin Dietrich, Marie-Josée Dumont, Tomislav Friščić* and Karine Auclair*

Figures:

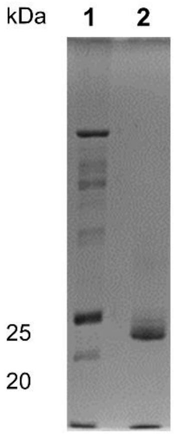

Figure S1: SDS-PAGE analysis (0.1 % SDS (w/v), 25 mM Tris) of commercial xylanase from *Thermomycec lanuginosus*. Lane 1 was used for the protein molecular weight markers and lane 2 for the xylanase. The gel was stained with Coomassie blue.

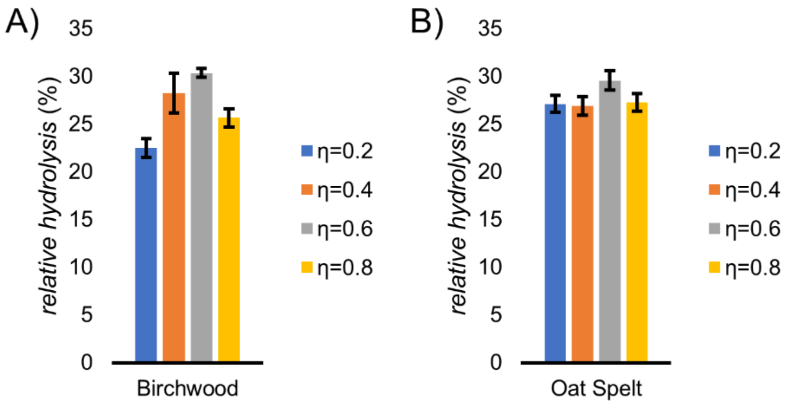

Figure S2: Optimization of the amount of water (reported as *η*, *i.e.* the volume of water added over the total amount of solid in μL/mg) for the hydrolysis of purified birchwood (A) or oat spelt (B) xylan by xylanase under milling conditions. The reaction mixtures contained 200 mg of birchwood or oat spelt xylan, 50 mg of the commercial xylanase mixture (*i.e.* 0.2 mg of protein, 0.08% loading w/w), and different volumes of water. The reaction was milled for 30 min at 30 Hz and room temperature, in a 14 mL volume teflon jar, containing two stainless steel balls of 7 mm diameter each.

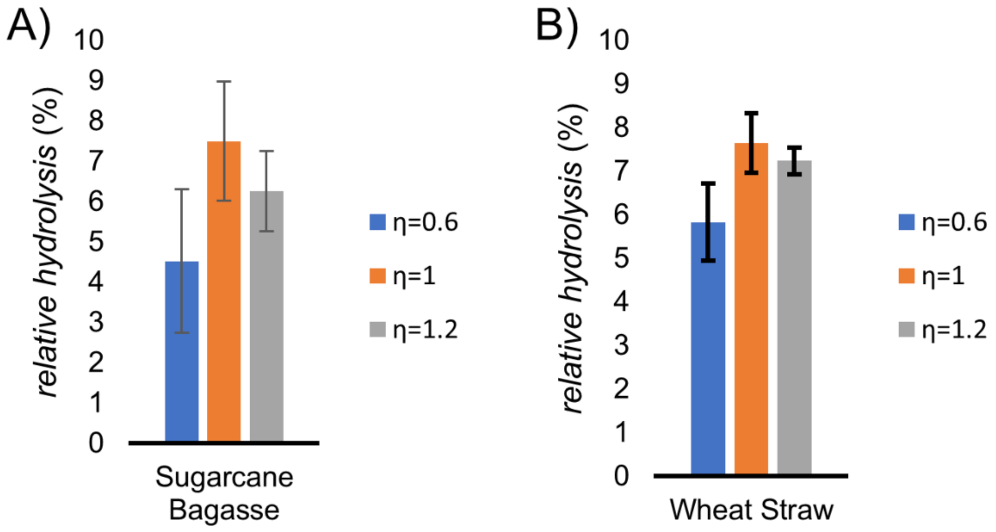

Figure S3: Optimization of the amount of water (reported as *η*, *i.e.* the volume of water added over the total amount of solid in μL/mg) for the hydrolysis of sugarcane bagasse (A) or wheat straw (B) xylan by xylanase under milling conditions. The reaction mixtures contained 400 mg of sugarcane bagasse or wheat straw, 200 mg of the commercial xylanase mixture (*i.e.* 0.8 mg of protein, 0.13% loading w/w), and different volumes of water. The reaction was milled for 30 min at 30 Hz and room temperature, in a 14 mL volume teflon jar containing two stainless steel balls of 7 mm diameter each.

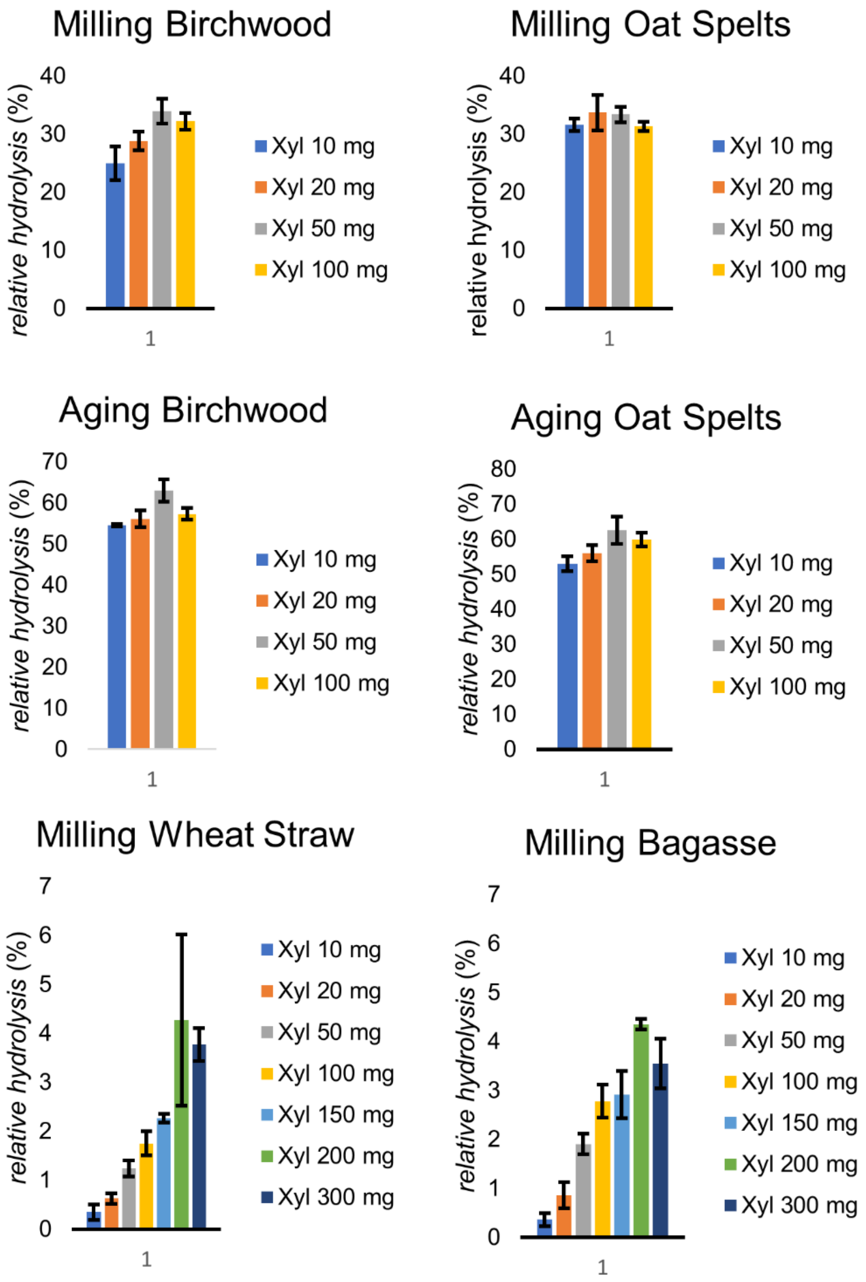

Figure S4: Optimization of the enzyme loading for the hydrolysis of birchwood xylan, oat spelt xylan, sugarcane bagasse or wheat straw by xylanase under milling only (30 min) or milling (30 min) followed by aging (72 h at 55°C). The reaction mixtures contained either 200 mg of xylan or 400 mg of biomass, 150 μL (for xylans) or 600 μL (for biomass) of water, and the various amounts of the commercial xylanase mixture listed (containing 0.4% protein w/w, *i.e.* 10 mg Xyl corresponds to 0.04 mg of protein, for a protein loading of 0.02% w/w with xylans and 0.04% w/w/ with biomass). The reaction was performed in a 14 mL volume teflon jar, containing two stainless steel balls of 7 mm diameter each.

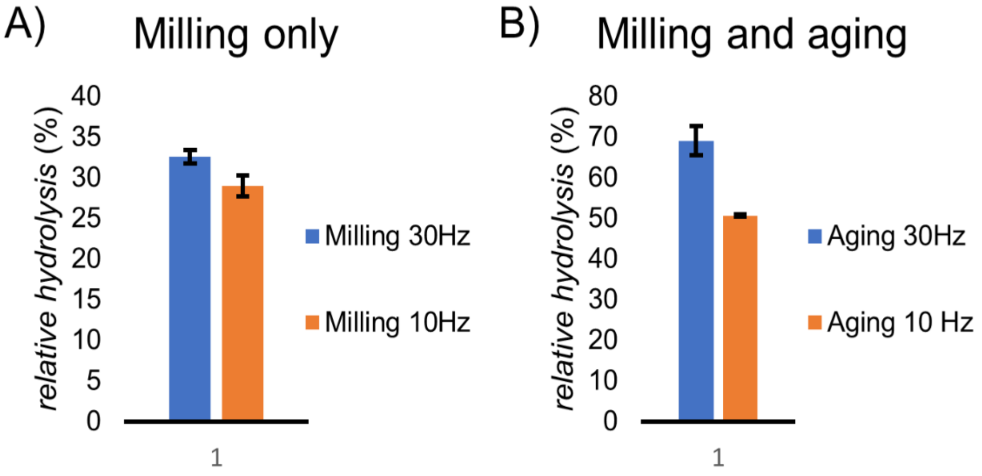

Figure S5: Effect of milling frequency (10 Hz or 30 Hz) on the xylanase-catalyzed hydrolysis of oat spelt xylan. The reaction was performed in a 14 mL volume teflon jar containing two stainless steel balls of 7 mm diameter each, and either milled for 30 minutes or milled (30 min) before aging for 72 h at 55°C. The reaction mixtures contained 200 mg of xylan, 150 µL of water, and 50 mg of the commercial xylanase mixture (*i.e.* 0.2 mg of protein, 0.03% loading w/w).

Tables

Table S1. Results of sugar analyses for selected transformations. Reactions contained 400 mg pre-milled substrate (1.5 g, 60 min) combined with 600 µL solution of NaN_3 (0.04% w/v) and CTec2 mg protein/g cellulose). Reactions were submitted to 12 cycles of 5 min milling at 30 Hz and 55 min aging at 55°C followed by another 1 h of aging at 55°C.

Process	Substrate	DNS yield (%)	Xylose yield (%)
Milling 30 min	Birchwood xylans	39	1
	Oat spelt xylans	41	1
	Sugarcane bagasse	12	0.3
	Wheat straw	8	0.1
Milling 30 min + Aging 72 h	Birchwood xylans	65	7
	Oat spelt xylans	68	6
	Sugarcane bagasse	85	5
	Wheat straw	95	3
RAging 12 h (12 cycles of 5 min milling + 55 min aging)	Birchwood xylans	47	5
	Oat spelt xylans	48	3
	Sugarcane bagasse	38	1
	Wheat straw	36	0.5

a) Based on the DNS assay. b) Measured by sugar analysis. Error is the standard deviation for triplicates.
